# Diagnostics of short tandem repeat expansion variants using massively parallel sequencing and componential tools

**DOI:** 10.1038/s41431-018-0302-4

**Published:** 2018-11-19

**Authors:** Rick H. de Leeuw, Dominique Garnier, Rosemarie M. J. M. Kroon, Corinne G. C. Horlings, Emile de Meijer, Henk Buermans, Baziel G. M. van Engelen, Peter de Knijff, Vered Raz

**Affiliations:** 1 Department of Human Genetics, Leiden University Medical Centre, Nijmegen, The Netherlands; 2 Department of Rehabilitation, Radboud University Medical Centre, Nijmegen, The Netherlands; 3 Department of Neurology, Donders Institute for Brain, Cognition and Behaviour, Radboud University Medical Centre, Nijmegen, The Netherlands

**Keywords:** Next-generation sequencing, Genetics research

## Abstract

Short tandem repeats (STRs) are scattered throughout the human genome. Some STRs, like trinucleotide repeat expansion (TRE) variants, cause hereditable disorders. Unambiguous molecular diagnostics of TRE disorders is hampered by current technical limitations imposed by traditional PCR and DNA sequencing methods. Here we report a novel pipeline for TRE variant diagnosis employing the massively parallel sequencing (MPS) combined with an opensource software package (FDSTools), which together are designed to distinguish true STR sequences from STR sequencing artifacts. We show that this approach can improve TRE diagnosis, such as Oculopharyngeal muscular dystrophy (OPMD). OPMD is caused by a trinucleotide expansion in the PABPN1 gene. A short GCN expansion, (GCN[10]), coding for a 10 alanine repeat is not pathogenic, but an alanine expansion is pathogenic. Applying this novel procedure in  a Dutch OPMD patient cohort, we found expansion variants from GCN[11] to GCN[16], with the GCN[16] as the most abundant variant. The repeat expansion length did not correlate with clinical features. However, symptom severity was found to correlate with age and with the initial affected muscles, suggesting that aging and muscle-specific factors can play a role in modulating OPMD.

## Introduction

STR sequences are widely spread over the genome and rarely directly related to inherited diseases [1]. However, some STRs, especially those where a single repeat unit encodes for an amino acid, are disease associated. A well-known example is a small group of TRE disorders, most of which are associated with hereditable disorders that often lead to neurological or neuromuscular symptoms [2–4]. These disorders can vary in symptom heterogeneity, the age of onset and can show a varied progression pattern, which together  complicate clinical diagnosis [2, 3]. Therefore, a definite diagnosis of TRE variants should be determined by a DNA-based genotyping. Accurate molecular diagnosis of short TRE disorders is technically challenging, as traditional DNA sequencing methods and PCR-based amplification methods impose limitations for TRE diagnosis [5–7].

STR amplification is prone to stutter artifacts, thereby creating noise in DNA sequencing data [8]. High coverage sequencing data in STR regions can be generated using massively parallel sequencing (MPS)  and combinning with statistical algorithms, the stutter characteristics and ratios can be accuratly determined [9]. This approach has been proven successful in STRs-based genotyping for forensic studies [10, 11]. Here we assessed whether MPS can be applied for accurate molecular diagnosis for TREs in Oculopharyngeal muscular dystrophy (OPMD).

OPMD (OMIM #164300) is caused by a short TRE in the first exon of the gene encoding for the polyadenylate-binding protein nuclear 1 (NG_008239.1 RefSeq gene on chromosome 14q11) [12]. The TRE in PABPN1 is an example of STRs located in coding regions. In the wild-type PABPN1 allele, the first methionine (ATG) is followed by a 10 alanine repeat (NM_004643: GCG[7]GCA[3]; named here as GCN[10], N represents A or G). A pathogenic PABPN1 was reported to have an 11 to 18 alanine range (GCN[11] to GCN[18], also referred as +1 to +8) [12]. The pathogenic protein is misfolded and aggregated, and consequently hampers cellular activities [3]. Most OPMD patients are heterozygous, with GCN[13] as the most abundant pathogenic expansion. Only a few homozygous cases were reported (GCN[13], GCN[11]) [12–15].

OPMD is a rare disorder (the prevalence is estimated at 1:100,000), but several studies suggested that OPMD is under and/or misdiagnosed [6, 7, 16, 17], due to unfamiliarity of clinicians, and due to the type of DNA test. Most often, Sanger sequencing is used for DNA diagnosis in OPMD. However, Sanger sequencing is not reliable for TRE sequencing because of low sequencing depth. Moreover, it can not accurately distinguish between two alleles that differ in length. The MPS will be suitable since this method sequences both alleles many times instead of resulting in one combined signal shown in an electropherogram. Current bioinformatics tools can provide an accurate analysis of STRs with massively parallel amplicon sequencing data [9]. Reliable and accurate genotyping of diseases caused by STRs is essential for an assessment of phenotype–genotype relations.

## Materials and methods

### Biofluid collection and DNA isolation

Patients were recruited through the Dutch neuromuscular database (Computer Registry of All Myopathies and Polyneuropathies: CRAMP). All participants signed informed consent and the study was approved by the local ethics committee. All patients visited the outpatient clinic at the Radboud University Medical Centre and were clinically examined on swallowing function, presence of ptosis and muscle weakness. Summary of clinical details are found in Table S1. Saliva was collected from patients and healthy controls for DNA analysis. Blood was collected from anonymous controls.

### Sample preparation

DNA was extracted from saliva that was collected from the OPMD subjects. The saliva was collected at room temperature, and RNAprotect® Saliva reagent (Qiagen) was added to the tube, according to the manufacturer’s instruction to stabilize RNA. Samples were kept in −80 °C prior to DNA isolation. DNA isolation was carried out with Trizol (Invitrogen) according to the manufacturer’s instructions. DNA quality was determined with the Qubit 4 fluorometer (ThermoFisher Scientific). Control samples were collected from anonymous subjects, from whom DNA was isolated from blood, as described in [18]. PCR was conducted with 1–5 ng DNA and 10 pmole *PABPN1*-specific primers targeting a 251 bp region including the GCN stretch in the first exon. Primers to *PABPN1* were designed with the Primer3 online tool (Table S2). These PABPN1-specific primers contained two different tails at the 5′ end of the primers, allowing a second PCR to incorporate the Illumina adapter sequences with barcoded primers (Table S2A) targeting the tails from the first PCR. In summary, PCR and library preparation is performed with two PCR reactions. First an amplification of PABPN1 using PABPN1_F and PABPN1_R primer set (Table S2), and a second PCR was performed with a multiplex barcoded primer set (Table S2B). Amplification performance of *PABPN1* was evaluated with three PCR kits: KAPA HiFi PCR (Roche), NEB Q5 kit (New England Biology) and AccuPrime™(ThermoFisher), according to the manufacturer’s instructions. Consistent results were obtained only with the KAPA HiFi PCR kit. PCR amplification was carried out with the following PCR program: 5 min denaturation at 98 °C, 30 times (10 s at 98 °C, 30 s at 60 °C, 30 s at 72 °C), and final extension for 3 min at 72 °C. PCR products were measured with 2100 Bioanalyzer High Sensitivity DNA Assay (Agilent Genomics). Subsequently, a clean-up was performed on the PCR products with Agencourt AMPure XP-beads (Beckman Coulter) according to the manufacturer’s instructions using a bead/sample ratio of 1.2×. Sample-specific barcoding was carried out in the second PCR with dual barcoded primers (Table S2B), using the same PCR program but for only 12 PCR cycles. Another PCR clean-up was performed with Agencourt AMPure XP-beads and the purified libraries were analyzed with the 2100 Bioanalyzer High Sensitivity DNA Assay (Agilent Genomics). In Fig. 2 at the left panel, we show the traces that were generated by the 2100 Bioanalyzer application prior to MiSeq sequencing.

### Sequencing and analysis

**Fig. 1:**
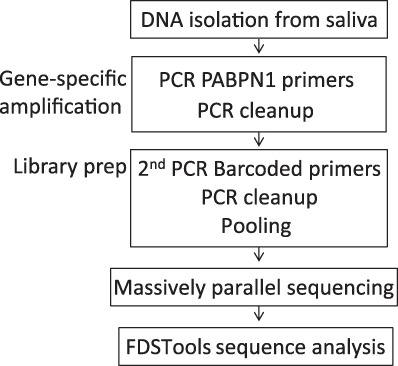
A flowchart of the DNAseq procedure

**Fig. 2:**
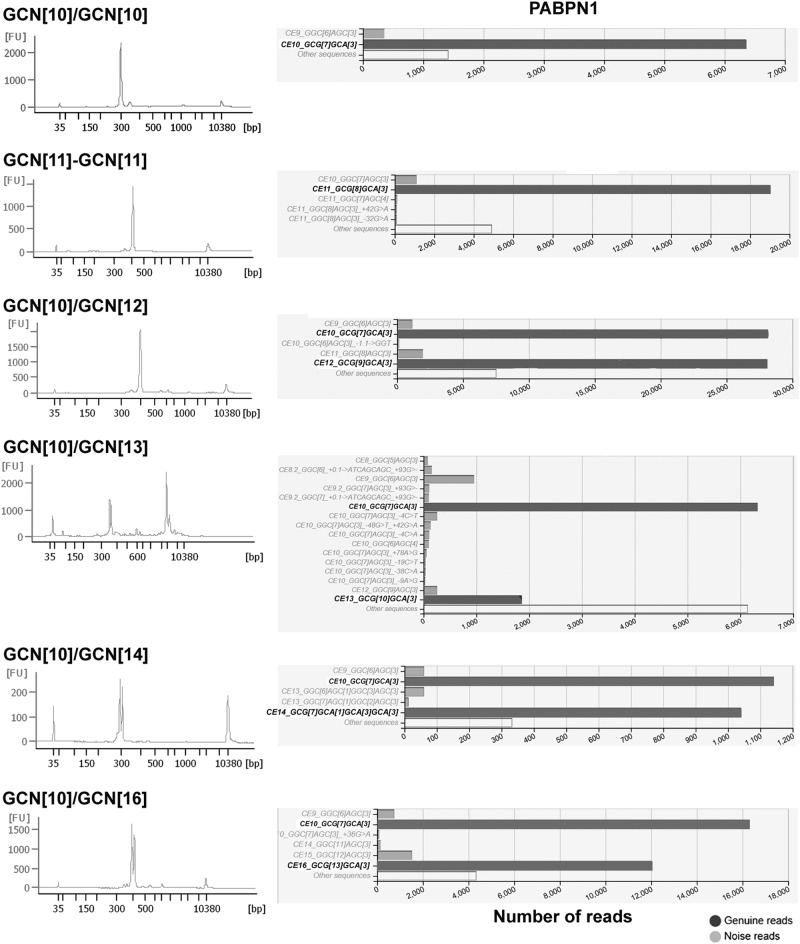
Examples of bioanalyses and FDSTools sequence results in OPMD and controls. Each row shows the result from a single subject. The left panel shows DNA histograms, which were generated by the DNA Bioanalyzer application. The right panel shows the graphical output of FDStools sequence analysis for an anonymous control and OPMD patients with five different GCN expansion lengths. The genuine alleles are highlighted in bold. The wild-type allele is depicted in black and the expanded allele in red. Non-genuine reads (PCR stutters, PCR-generated point mutations and sequencing errors) are shown in gray

Two times 300 bp paired-end MPS data were generated using the MiSeq^®^Sequencer (Illumina) with the Miseq reagents v3 600 cycles kit. A summary of the procedure is shown in Fig. 1. DNAseq analysis, sample filtering and data interpretation were carried out with FDSTools as detailed in [9]. In brief: Read-pairs were merged after alignment. Merged reads were mapped to *PABPN1* using annotation.*TSV*, which counts and summarizes all sequenced reads that include the PABPN1 primer sequences (with a mismatch allowance of 10%). All reads within a sample with a frequency of one were filtered out and clustered into a single “other sequences”. A distinction between noise and true allelic sequences was made from the FDSTools visualizations. The allele with most reads and proper sequence structure was considered as a genuine allele in the sample. Heterozygosity was determined by the second most frequent allele that had a sequencing depth above the minimum threshold (we set it for *N* = 10). We found that the depth of the pathogenic allele was on average 56.5% of the wild-type allele in a Dutch OPMD cohort. PCR and sequencing artifacts like STR-stutter and point mutations can be recognized by FDSTools when a database with known genotypes is supplied. In this study manual analysis of the FDStools visualizations was used for genotyping, since a known OPMD database is currently lacking. The data was then uploaded into LOVD.nl, creating the first OPMD variant database. Sequences and corresponding read depth were used to distinguish PCR and sequencing artefacts from genuine alleles. The “Other sequences” that are shown in Fig. 2 include all reads that did not meet the criteria to be shown in the FDSTools figures. These criteria are user depended and were set to a minimum of 10 reads and a minimum of 0.5% from the highest PAPBN1 allele in the sample. DNA diagnostic results are found in Table S1. Statistical tests were made in Prism 7.

## Results and discussion

**Fig. 3:**
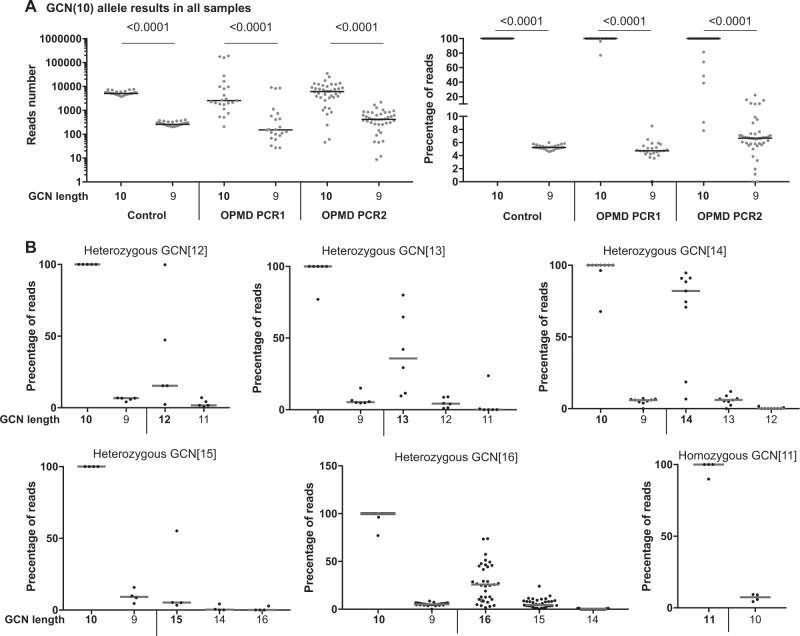
Stutters in trinucleotide GCN expansion. **a** Analysis of the wild-type allele (GCN[10]) in control and OPMD patients. The number of reads (left panel) or the percentage of reads with respect to the highest allele in the sample (right panel) are plotted against GCN length results. In the control group, results are from one PCR experiment. In the OPMD group, results are from two experiments: 20 amplification cycles (PCR1) or 40 amplification cycles (PCR2). The stutter percentage was significantly higher in the second experiment. Genuine allele length is depicted in bold and stutter allele length is in plain text. *P*-value was calculated by the Student’s *T*-test. **b** Analysis of sequencing results in OPMD subjects from two experiments. Plots show percentage of reads with respect to the highest allele in the sample for subjects with the same genotype (heterozygous: GCN[12], GCN[13], GCN[14], GCN[15], GCN[16], and homozygous GCN[11]). In each plot, the left side shows the wild-type GCN[10] and corresponding stutter GCN[9], the right side shows the expanded allele and its corresponding stutters. Genuine GCN length is depicted in bold and stutters are plain. Each dot represents an individual. Median is marked with a black line

DNA from 50 putative OPMD patients (Table S1) and 25 anonymous healthy controls was first amplified using PABPN1-specific primers, and subsequently amplified with sample-specific barcoded Illumina compatible primers (Table S2). Results of multiplex sequencing in sufficient depth was then analysed using FDSTools, which identifies PCR stutters, and subsequently determines genuine alleles that were then manually considered for genotyping (Fig. 1). FDSTools is an open-source software package that can interactively visualize amplicon sequencing data. When FDStools is supplied with deep sequencing data from a known OPMD database, it can create a noise database and use this to determine and visualize systemic noise like stutter alleles and sequencing artefacts in the amplicon sequencing data. Moreover, FDStools is able to automate allele calling [9]. In this study, allele calling was manually made from the FDStools visualization output, since such an MPS database for PABPN1 variants is not yet available. The FDSTools output shows the statistics and number of reads for each sequence per subject (examples are in Fig. 2). The proportion of reads per sequence is calculated from the most frequent sequence found in the sample (denoted the most frequent sequence as 100%). All other sequences are denoted with a percentage relative to this depth (examples are in Fig. 3). Allele calling is made by the two most frequent sequences.

Prior to sequencing, PCR products were analyzed with the Agilent 2100 Bioanalyzer (Fig. 2, left panel). Two peaks, corresponding to the wild-type and expanded alleles in DNA from heterozygous patients with long expansion variants GCN[13–16], were found in the DNA Bioanalyzer output (Fig. 2, left panel). MPS sequencing and FDSTools analysis also showed two alleles in patients carrying the GCN[13–16] variants (Fig. 2, right panel). This indicates heterozygosity in those patients. In control subjects only a single peak was found with the DNA Bioanalyzer (Fig. 2, left panel), and accordingly only one genuine allele (GCN[10]) was identified using MPS sequencing and FDSTools analysis (Fig. 2, right panel). The short expansion variants (GCN(11–12) were detected only with MPS and FDSTools analysis (Fig. 2, right panel). The resolution of the DNA Bioanalyzer was insufficient to separate between GCN[10] and GCN[12] (Fig. 2, left panel). Additionaly,  only GCN[11] expansion was found in two patients, indicating that they are homozygous carriers (Fig. 2, right panel). Consistently, a manual control of the FDSTools output clearly distinguished between the genuine allele and stutters or PCR errors. Together, this procedure suggests that heterozygosity of familial GCN[13–16] expansion variants could be accurately determined by the fast and simple DNA Bioanalyzer procedure. Identification of exact repeat lengths and shorter expansion variants require MPS sequencing at sufficient depth and proper sequence analysis to discriminate short stutters from the genuine alleles.

To assess the prevalence of stutters we focused on the GCN[10] allele in all samples (controls and OPMD) and found a common shorter trinucleotide stutter (−1) (Fig. 3a). The (−1) stutter was found in all samples regardless of genotype, DNA extraction procedure, or the source of biofluid (Fig. 3a). To assess reproducibility of the results, the procedure was repeated twice. In the first experiment the first target-specific PCR amplification was carried out with 20 cycles, and in the second experiment with 40 cycles. Overall, genotyping was consistent in both experiments. However, a higher percentage of stutter was found in the 40-cycle PCR protocol (Fig. 3a). In addition, higher stutter percentage was also found in samples with low reads, indicating that reliable genotyping requires deeper sequencing  output (between 5000 and 10,000 reads per sample). However, a sequencing depth around 1000 reads per sample was also found to be reliable. We found that the complexity of stutters increased with the longer expansion variants (Fig. 3b). In patients with the GCN[16], allele stutters were more abundant compared with the shorter expansions. Most often stutters were shorter (−1 to −3) (Fig. 3b). In only one case, (GCN[15]) a + 1 stutter was found (Fig. 3b), but that +1 stutter could not be reproduced.

Stutters are common artefacts when amplifying repetitive regions [9, 19]. Accurate diagnosis requires discrimination of stutters from the genuine alleles, which is not possible with traditional Sanger DNA sequencing [20]. Moreover, Sanger sequencing does not accurately differentiate between two alleles of different lengths, and thus it is not suitable to determine heterozygosity. Overall, most diagnosis of trinucleotide expansion variants is carried out with a Sanger sequencing [11, 21]. In contrast to Sanger sequencing, MPS can generate sequencing reads of sufficient depth allowing overcoming sequencing errors. In combination with FDSTools STR stutters can be identified, and the genuine alleles can be discriminated from stutters. Apart from discriminating genuine alleles from stutters we show that optimization of the PCR protocol can significantly reduce stutter formation in PCR.

**Fig. 4:**
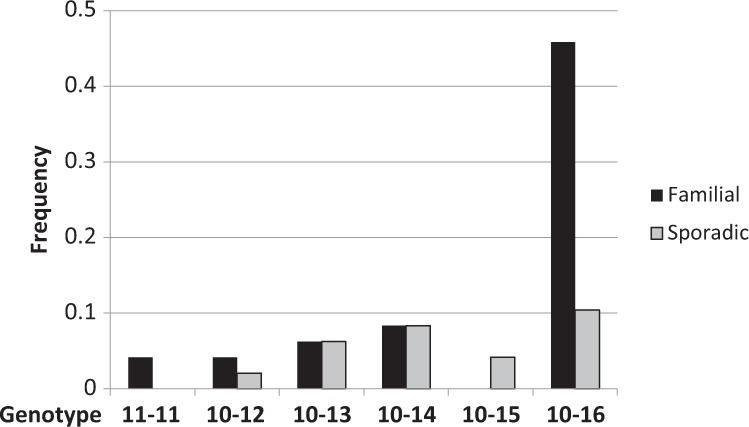
The frequency of GCN expansion length in the Dutch OPMD patients. Bar chart shows the frequency of GCN expansion variants in familial and sporadic OPMD. The GCN[16] expansion is enriched in the Dutch familial OPMD. The GCN[15] expansion is found only in sporadic OPMD, the GCN[11] expansion is only familial

From the 50 OPMD subjects that were included in this study, two subjects from OPMD families had muscle weakness complaints but did not have a trinucleotide expansion variant. From the 48 genetically confirmed OPMD subjects, a familial GCN expansion was found for 35 subjects (73%). The other 13 subjects were either non-familial (sporadic), or unknown familial genetic background (Table S1). The range of GCN expansions varied between GCN[11] to GCN[16] (Table S1). Heterozygosity for GCN[10]/GCN[16] was most abundant among the familial subjects, but no specific preference of expansion variants was found in the sporadic cases (Fig. 4). However, the small number of subjects limits statistical tests.

In a previous study, we used Sanger sequencing for DNA diagnosis [22]. Nine subjects were included in both studies, and discrepancies between GCN expansion length were found for only three patients. Two non-familial patients were diagnosed as GCN[13] with Sanger sequencing, but with MPS were found to have GCN[14]. One patient was diagnosed with GCN[14] with Sanger sequencing, but GCN[16] was found with the MPS. Four family members of this patient also have GCN[16], which supports an accurate diagnosis with MPS. The higher prevalence of GCN[16] variant in this Dutch OPMD cohort is in contrast to other national studies. Those studies showed that the GCN[13] variant is the most abundant [23]. In a French OPMD cohort, heterozygous GCN[13] was the most abundant, whereas the GCN[16] variant was rare [14]. In Mexico, the GCN[15] variant is the most abundant (65% (66 out of 102). Heterozygous compound variants were reported very rarely [14, 24, 25]. As those were not confirmed by other procedures, it could be a mistake of the Sanger results. Homozygous GCN[11] were found in both French and Dutch studies. OPMD patients with the GCN[11] variant are not common [23], but heterozygous GCN[11] was reported in the general population, estimated as 0.05–2% [17, 26]. As those studies were carried out with the Sanger sequencing, this non-pathogenic polymorphism should be confirmed using MPS with FDSTools analysis. Moreover, we found major differences in clinical severity between the two GCN[11] homozygous subjects, albeit  a close family relationship and only one year difference in age. The brother has swallowing dysfunction, severe ptosis for which an unsuccessful surgery was tried, and severe leg muscle weakness resulting in wheelchair dependence. In contrast, his sister had minor swallowing dysfunction in clinical testing without other signs of weakness. She underwent an eyelid correction in the past. Together, it is unclear why certain heterozygous GCN[11] cases develop muscle weakness later in life [14 and this study], but others have severe muscle weakness (this study). This supports our hypothesis that secondary aging-associated triggers are involved in symptom manifestation in OPMD [27].

We then investigated a correlation between genetics and clinical or demographic features (Table 1). No significant correlation was found between GCN expansion length and clinical features in OPMD patients (Table 1). This observation is consistent with a study assessing a phenotype–genotype relation in a Spanish OPMD study [28], and with our previous study in a smaller Dutch OPMD cohort [22]. However, two other studies suggested a weak correlation between GCN expansion length and age of onset [14, 24]. In addition, in this study, we found a significant correlation between age and symptom severity (*r* = 0.471, *p*-value 0.001), which is consistent with our previous study [22]. In addition, in this Dutch OPMD cohort we also found a significant correlation between the initial features (ptosis, dysphagia, or leg weakness) and symptom severity (*r* = 0.482, *p*-value 0.001). Patients with initial leg weakness symptoms were more severe. It is yet obscure whether in OPMD, alanine expansion length affects muscle weakness severity. Recent studies suggested that additional factors, other than the known genotype, could modulate disease severity [29]. Our previous studies suggested that aging factors could affect symptom initiation [27]. In addition, the correlation between initial clinical features and severity suggests that muscle-specific factors could modulate symptom severity.

**Table 1:** Pearson correlation in confirmed exp*PABPN1* carriers between age, family, GCN length, and symptom severity

		Pearson *r*					
		Age	Gender	GCN expansion length	Symptom severity	Diagnosis duration	First feature
*p*-value	Age		0.132	−0.237	**0.471**	0.231	−0.002
	Gender	0.372		−0.021	0.032	0.039	0.184
	GCN expansion length	0.105	0.886		0.080	−0.016	0.166
	Symptom severity	**0.001**	0.827	0.589		0.052	**0.482**
	Diagnosis duration	0.126	0.801	0.919	0.736		−0.126
	First feature	0.992	0.236	0.289	**0.001**	0.440	

Symptom severity: mild (one muscle is affected); severe (two or more muscles are affected). First features: clinical features: ptosis, dysphagia or leg weakness. *p*-value was calculated by the Pearson test. Sinigicant correlations are in bold.

Several studies suggested that OPMD is under-diagnosed [6, 7], in part, because OPMD is aging associated [27], thus it is likely that the patients with a late onset are misdiagnosed or not subjected to DNA diagnosis. In addition, OPMD genetics is variable: most reported patients are heterozygous dominant [14, 23, 30]. Homozygous dominant patients are very rare [14, 15]. Few recessive OPMD cases were also reported [31, 32]. The variability of symptoms and progression in OPMD could also contribute to misdiagnosis. We present a procedure for accurate genotyping of trinucleotide expansion regions. Advances in DNA sequencing reveal that missing alleles (deleted or allelic dropouts) can lead to misdiagnosis [33]. With MPS we still cannot recognize those cases and thus cannot exclude the possibility that those cause OPMD. Moreover, allelic dropouts were recently recognized in trinucleotide expansion repeats using Pac-Bio sequencing [20]. For accurate OPMD diagnosis and an assessment of the spectrum of OPMD genetics, additional studies are required.

## Electronic supplementary material

Supplementary Tables
