# Supplementary material for: Diagnostics of short tandem repeat expansion variants using massively parallel sequencing and componential tools
Source: Eur J Hum Genet. 2018 Nov 19;27(3):400–7. doi: 10.1038/s41431-018-0302-4 (PMC6460572; doi:10.1038/s41431-018-0302-4)
Supplement: Supplementary file 1 — Supplementary Tables [file 41431_2018_302_MOESM1_ESM.docx]

**Supplementary tables**

**Table S1: Clinical summary and genotyping results**

| **Patient nr.** | **Family nr.** | **GCN** | **Age** | **gender^$^** | **Symptom severity^*^** | **Diagnosis duration (yrs)** | **First feature** |
| --- | --- | --- | --- | --- | --- | --- | --- |
| P8 | 1 | 12 | 50 | M | asymp |  | ND |
| P10 | 1 | 12 | 54 | F | asymp |  | ND |
| P3 | 2 | 16 | 63 | M | mild | 5 | Ptosis |
| P15 | 2 | 16 | 61 | M | mild | 6 | Dysphagia |
| P16 | 2 | 16 | 54 | F | mild | 3 | Dysphagia |
| P23 | 2 | 16 | 59 | F | mild | 7 | Ptosis |
| P48 | 2 | 16 | 57 | F | asymp |  | ND |
| P49 | 2 | 10 | 66 | F | asymp |  | ND |
| P12 | 3 | 16 | 57 | M | mild | 7 | Dysphagia |
| P35 | 3 | 16 | 60 | F | mild | 20 | ND |
| P40 | 3 | 16 | 55 | F | mild | 55 | ND |
| P45 | 3 | 16 | 64 | F | mild | 5 | Dysphagia |
| P36 | 4 | 14 | 55 | F | mild | 10 | Dysphagia |
| P38 | 4 | 14 | 51 | M | mild | 8 | Ptosis |
| P42 | 5 | 14 | 59 | M | mild | 9 | Dysphagia |
| P46 | 5 | 14 | 64 | F | mild | 24 | Dysphagia |
| P2 | 6 | 16 | 50 | M | severe | 13 | ND |
| P13 | 6 | 10 | 55 | F | mild | 7 | Ptosis |
| P4 | 7 | 16 | 58 | M | mild | 13 | Dysphagia |
| P39 | 7 | 16 | 53 | M | mild | 4 | Ptosis |
| P5 | 8 | 16 | 75 | F | severe | 15 | Legs |
| P6 | 8 | 16 | 50 | F | mild | 1 | Dysphagia |
| P20 | 8 | 16 | 63 | M | mild | 3 | Ptosis |
| P21 | 8 | 16 | 63 | F | mild | 4 | Ptosis |
| P50 | 8 | 16 | 49 | M | asymp | 49 | ND |
| P25 | 9 | 16 | 55 | F | mild | 7 | Dysphagia |
| P30 | 9 | 16 | 79 | F | severe | 42 | Ptosis |
| P31 | 9 | 16 | 53 | M | mild | 6 | Dysphagia |
| P41 | 9 | 16 | 52 | F | severe | 8 | Legs |
| P47 | 9 | 16 | 62 | M | mild | 7 | Ptosis |
| P32 | 10 | 13 | 44 | F | mild | 2 | Legs |
| P33 | 10 | 13 | 73 | F | severe | 23 | Dysphagia |
| P37 | 10 | 13 | 64 | F | mild | 16 | Ptosis |
| P18 | 11 | 11 | 75 | F | mild | 25 | Dysphagia |
| P24 | 11 | 11 | 74 | M | severe | 7 | Ptosis |
| P1 |  | 15 | 68 | M | severe | 28 | Ptosis |
| P7 |  | 13 | 62 | F | mild | 8 | Dysphagia |
| P9 |  | 16 | 51 | F | mild | 4 | Dysphagia |
| P11 |  | 16 | 54 | F | mild | 5 | Dysphagia |
| P14 |  | 16 | 49 | M | mild | 5 | Dysphagia |
| P17 |  | 16 | 73 | M | severe | 20 | Legs |
| P19 |  | 14 | 63 | M | mild | 7 | Legs |
| P22 |  | 12 | 77 | F | mild | 4 | Dysphagia |
| 26 |  | 14 | 59 | M | mild | 59 | ND |
| P27 |  | 13 | 62 | M | mild | 7 | Ptosis |
| P28 |  | 16 | 77 | F | mild | 29 | Ptosis |
| P29 |  | 13 | 59 | M | mild | 11 | Ptosis |
| P34 |  | 15 | 62 | M | mild | 8 | Ptosis |
| P43 |  | 14 | 67 | M | mild | 17 | Ptosis |
| P44 |  | 14 | 67 | F | severe | 20 | Dysphagia |
| Gender: M=male; F= female | | | | | | | |
| Symptom severity: mild (one muscle is affected); severe (two or more muscles are affected); asymptomatic (asymp, no muscles are affected). | | | | | | | |

**Table S2**

**A: *PABPN1*-specific primers**

|  | | | **Tail** | | | ***PABPN1*** | **%GC** | **Product length** | | |  |  |  |
| --- | --- | --- | --- | --- | --- | --- | --- | --- | --- | --- | --- | --- | --- |
| *PABPN1*_F | | GATGTGTATAAGAGACAG | | | GCAGGCAGCTTGACTAATGA | | 78 | 251 | | |  |  |  |
| *PABPN1*_R | | CGTGTGCTCTTCCGATCT | | | CTCCTCAGGCTCCAGTTCCT | |  |  |  |  |  |  |  |
|  |  | | |  |  |  |  |  |  |  |  |  |  |
| **B: Barcoded primer sets** | | | | | | | | |  | | |  |  |
| I7 primer sequence : CAAGCAGAAGACGGCATACGAGAT\|index-i7\|GTGACTGGAGTTCAGACGTGTGCTCTTCCGATCT  I5 primer sequence: AATGATACGGCGACCACCGAGATCTACAC\|index-i5\|TCGTCGGCAGCGTCAGATGTGTATAAGAGACAG   \| i7 \| i5 \| \| --- \| --- \| \| AAGAGGCA \| TATAGCCT \| \| AAGAGGCA \| ATAGAGGC \| \| ATTACTCG \| TATAGCCT \| \| ATTACTCG \| ATAGAGGC \| \| ATTACTCG \| GGCTCTGA \| \| ATTACTCG \| AGGCGAAG \| \| ATTACTCG \| GTACTGAC \| \| TCCGGAGA \| TATAGCCT \| \| TCCGGAGA \| ATAGAGGC \| \| TCCGGAGA \| CCTATCCT \| \| TCCGGAGA \| GGCTCTGA \| \| TCCGGAGA \| AGGCGAAG \| \| CGCTCATT \| ATAGAGGC \| \| CGCTCATT \| AGGCGAAG \| \| CGCTCATT \| CAGGACGT \| \| CGCTCATT \| GTACTGAC \| \| GAGATTCC \| TATAGCCT \| \| GAGATTCC \| CCTATCCT \| \| GAATTCGT \| TATAGCCT \| \| GAATTCGT \| ATAGAGGC \| \| GAATTCGT \| CCTATCCT \| \| GAATTCGT \| TAATCTTA \| \| GAATTCGT \| CAGGACGT \| \| GAATTCGT \| GTACTGAC \| \| ATTCAGAA \| TATAGCCT \| \| ATTCAGAA \| ATAGAGGC \| \| ATTCAGAA \| CCTATCCT \| \| ATTCAGAA \| GGCTCTGA \| \| ATTCAGAA \| TAATCTTA \| \| ATTCAGAA \| CAGGACGT \| \| ATTCAGAA \| GTACTGAC \| | | | | | | | | |  |  |  |  |  |

Table lists the gene specific forward and reverse primers for *PABPN1 (A). Each* PABPN1 specific *primer has a* tail on the 5’end , those primers were used in PCR1. The tail functions as a docking site for to the dual index barcoded primer sets: i7 (left) and i5 (right) in PCR2 (B). Sample indexing was carried out by a dual index combination. The tail sequence is underlined in B.
